# Memantine/Rosuvastatin Therapy Abrogates Cognitive and Hippocampal Injury in an Experimental Model of Alzheimer's Disease in Rats: Role of TGF-β1/Smad Signaling Pathway and Amyloid-β Clearance

**DOI:** 10.1007/s11481-024-10159-1

**Published:** 2024-12-21

**Authors:** Esraa F. Zidan, Nesrine S. El-Mezayen, Safaa H. Elrewini, Elham A. Afify, Mennatallah A. Ali

**Affiliations:** 1 Department of Pharmacology and Therapeutics, Faculty of Pharmacy, Pharos University in Alexandria, Alexandria, Egypt; 2 Department of Clinical Pharmacology, Faculty of Medicine, Alexandria University, Alexandria, Egypt; 3 Department of Pharmacology and Toxicology, Faculty of Pharmacy, University of Alexandria, Alexandria, Egypt; 4 Department of Pharmacology and Toxicology, PharmD Program, Egypt-Japan University of Science and Technology (E-JUST), Alexandria, Egypt

**Keywords:** Alzheimer, β-amyloid, Blood-brain barrier transporters, MicroRNA, TGF-β

## Abstract

Alzheimer’s disease (AD) is a multifactorial neurodegenerative disorder of complex pathogenesis and multiple interacting signaling pathways where amyloidal-β protein (Aβ) clearance plays a crucial role in cognitive decline. Herein, the current study investigated the possible modulatory effects of memantine/ rosuvastatin therapy on TGF-β1/p-Smad/p21 signaling pathway and their correlation to the blood brain barrier transporters involved in Aβ-clearance and microRNAs as a novel molecular mechanism in AD treatment. AD was induced by a single intracerebroventricular streptozotocin injection (ICV-STZ, 3 mg/kg) in rats and drug therapy was continued for 28 days after AD induction. Efficacy was monitored by applying a battery of behavioral assessments, as well as biochemical, histopathological, molecular and gene expression techniques. The upregulated TGF-β1-signaling in the untreated rats was found to be highly correlated to transporters and microRNAs governing Aβ-efflux; ABCA1/miRNA-26 and LRP1/miRNA-205 expressions, rather than RAGE/miRNA-185 controlling Aβ-influx; an effect that was opposed by the tested drugs and was found to be correlated with the abolished TGF-β1-signaling as well. Combined memantine/rosuvastatin therapy ameliorated the STZ evoked decreases in escape latency and number of crossovers in the Morris water maze test, % spontaneous alternation in the Y-maze test, and discrimination and recognition indices in the object recognition test. The evoked behavioral responses were directly related to the β-amyloid accumulation and the alteration in its clearance. Additionally, drug treatment increased brain glutathione and decreased malondialdehyde levels. These findings were histopathologically confirmed by a marked reduction of gliosis and restoration of neuronal integrity in the CA1 region of the hippocampus of the AD rats. These findings implicated that the memantine/rosuvastatin combination could offer a new therapeutic potential for AD management by abrogating the TGF-β1/p-Smad2/p21 pathway and regulating Aβ-clearance.

**Graphical Abstract:**

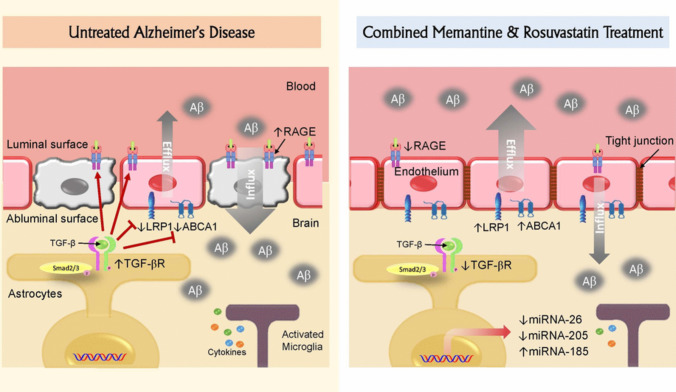

**Supplementary Information:**

The online version contains supplementary material available at 10.1007/s11481-024-10159-1.

## Introduction

Alzheimer’s disease (AD) is a progressive neurodegenerative brain disorder that is considered the most common cause of dementia and mental deterioration in the elderly population (Alzheimer's Association, 2023). The pathological hallmarks of AD are the intracellular neurofibrillary tangles (NFTs) and extracellular amyloidal β protein (Aβ) deposits, which lead to the formation of senile plaques in multiple brain regions involved in learning, memory and emotional behaviors (Kumar et al., 2015). Its pathophysiology is based on the tau and Aβ hypothesis. The tau protein, a microtubule-associated protein, stabilizes the neuronal cytoskeleton via the interaction between tau and tubulin through a crucial dynamic process regulated by its phosphorylation state. Hyperphosphorylation of tau protein by enzymes, including GSK-3β, leads to the disintegration of microtubules and the formation of the fibrillary aggregates NFTs, eventually resulting in neuronal damage. Furthermore, Aβ hypothesis defines the extracellular plaques, which are deposits of fibrils and amorphous aggregates of Aβ protein, originated from amyloid precursor protein (APP) (Aillaud and Funke, 2022). In AD, there is an imbalance between production and clearance of Aβ, leading to the accumulation of Aβ plaques in the brain. This accumulation is thought to trigger neuroinflammation and immune responses, leading to further neuronal damage. The microglia, found proximate to Aβ plaques, play a significant role in innate immunity and the expression of cytokines and chemokines. However, during AD pathogenesis, the activation of the immune system and microglia is unable to confine the Aβ plaques, exacerbating NET formation (Dubey et al., 2018a).

Although the neuropathological features of AD are recognized and multitude crosstalking trajectories such as vascular alteration, insulin resistance, neuroinflammation and oxidative stress are attributed to its pathogenesis, the specific underlying molecular mechanisms of AD remain ambiguous (Gadhave et al., 2021).

As mentioned, the Aβ protein deposits are delineated as the core of AD pathophysiology. Reportedly, prolonged monitoring of patients with high Aβ levels showed a decline in attention and episodic memory, including verbal list learning, visual pattern separation, and paragraph learning (Harrington et al., 2017). These findings might be attributed to the early predilection for Aβ-induced cell death in the medial temporal lobes (Sperling et al., 2011). These changes might be also associated with a decline in global cognition, visuospatial and executive functions (Baker et al., 2017). In addition, a previous study reported a causal relationship between the reduction of Aβ plaque and diminution in cognitive and functional skills in patients with AD (Pang et al., 2022).

It is hypothesized that Aβ accumulation ascends from a failure of clearance rather than over-production (Hardy and Selkoe, 2002). The blood-brain barrier (BBB) provides a large surface area for influx and efflux mechanisms into and out of the brain, respectively. Different transporters and receptors expressed on the two sides of the membrane; the luminal side facing the blood circulation, and the abluminal side facing the brain parenchyma, have been implicated to play crucial roles in Aβ clearance from brain (Madadi et al., 2019). The receptor for advanced glycation end products (RAGE), which belongs to the immunoglobulin family is expressed on the luminal surface of brain vessels. RAGE is a multi-ligand receptor that can bind to many ligands including Aβ causing internalization of its soluble monomeric isoforms (Malherbe et al., 1999). Since RAGE mediates Aβ entrance to the brain, RAGE upregulation offsets Aβ accumulation and cognitive impairment (Ma et al., 2017). Low-density lipoprotein receptor (LDLR) family are cell surface receptors implicated in receptor-mediated endocytosis and their over-expression promotes Aβ clearance (Kim et al., 2009). One of LDLRs is LDL receptor–related protein 1 (LRP1); an abluminal receptor that mediates Aβ transport across the BBB and brain-to-blood Aβ clearance (Zlokovic et al., 2010). Impaired recognition memory and reduced clearance of Aβ through BBB was observed upon using LRP1 oligodeoxynucleotide antisense in mice (Jaeger et al., 2009). ATP‐binding cassette (ABC) transporters are one of the most common transmembrane proteins that actively transport substrates across membranes; ABC transporters, especially ABCA1, ABCA7, ABCB1, ABCG2, and ABCG4, are involved in Aβ clearance. Among which, ABCA1 is expressed on the abluminal side of the BBB and functions to transport cholesterol and phospholipids to ApoE for creating high-density lipoproteins (Akanuma et al., 2008). Cumulative evidence revealed that ABCA1 indirectly expedites Aβ clearance through ApoE lipidation in the brain (Corona et al., 2016). Further, mutated ABCA1 was found to be associated with a higher risk of AD (Nordestgaard et al., 2015). This function is closely similar to that of ABCA7, which was previously reported to promote cellular cholesterol efflux and inhibit Aβ production (Chan et al., 2008).

MicroRNAs (miRNAs) are small non-coding RNAs that serve as essential post-transcriptional regulators of gene expression. Among genes regulated by miRNAs are those encoding BBB transporters governing Aβ clearance; RAGE, LRP1 and ABCA1 are regulated by miR-185, miR-205 and miR-26, respectively (Song and Bu, 2009; Sun et al., 2012; Jing et al., 2015). In AD, these miRNAs are dysregulated leading to altered transporters protein expression, which in turn results in a pathogenic signaling network triggering the imbalance between Aβ peptide synthesis and clearance (Madadi et al., 2019). These derangements are directly linked to the cognitive and functional decline in AD (Harrington et al., 2017; Pang et al., 2022).

Transforming growth factors (TGFs)/Smads pathway is also among the postulated trails in AD pathogenesis (von Bernhardi et al., 2015). TGF-β1 is a pleiotropic cytokine, which acts as a potent regulator of neuroinflammation and cytotoxicity. Nevertheless, in ageing, high levels of TGF-β1 promote neuroinflammation rather than its canonical role in attenuating immune responses (Yousef et al., 2015). Furthermore, chronically activated microglia showed a reduced response to modulation by TGF-β secreted by astrocytes (Cornejo and von Bernhardi, 2016), facilitating a cytotoxic activation of microglia and potentiating microglia-mediated neurodegeneration. These derangements have been linked to neuronal damage, cognitive impairment, and AD (von Bernhardi et al., 2015).

Currently only few approved drug therapies are available to provide symptomatic treatments for AD, among which memantine. Memantine is a low to moderate affinity N-methyl-D-aspartate (NMDA) receptor antagonist, approved for the management of moderate to severe AD. Memantine blocks glutamate and subsequently prevents excessive excitotoxicity and neuronal cell death, halting the cognitive deficiencies seen in AD (Yiannopoulou and Papageorgiou, 2020). Its proposed neuromodulatory mechanisms include antiapoptotic (Song et al., 2015), antioxidant and anti-inflammatory effects, as well as a reduction of microglial activation and an increase of the release of neurotrophic factors from astroglia (Wu et al., 2009). However, a deeper insight into its effects on the crosstalking trails contributing to AD pathogenesis is still indispensable. In addition, monotherapy in AD treatment possesses multiple limitations regarding safety, efficacy, and disease modification, which necessitates the combination therapy (Kabir et al., 2020).

Rosuvastatin is an antihyperlipidemic agent that competitively inhibits hydroxymethylglutaryl-coenzyme A (HMG-CoA) reductase, the rate-limiting step in cholesterol biosynthesis, at a high affinity (Luvai et al., 2012). Studies reported that high cholesterol might influence the β-/γ-secretase activity, leading to Aβ generation from APP, reduce the flux of APP via the non-amyloidogenic pathway, and affect several non-amyloid factors such as tau metabolism or local inflammation, which are correlated to AD pathogenesis (Gamba et al., 2019). Herein, the current study tested the hypothesis that modulation of TGF-β1/p-Smad/p21 signaling by memantine, rosuvastatin and their combination and their effect on BBB transporters that govern Aβ clearance and their regulating miRNAs may provide therapeutic potential against AD. Combination of behavioral, oxidative capacity, molecular and brain histopathological studies are applied in an STZ induced AD like model in rats to explore the possible modulation of downstream TGF-β/p-Smad/p21 signaling pathway, as a novel molecular target of both drugs in the management of AD.

## Materials and Methods

### Animals

Forty adult male Sprague-Dawley rats weighing approximately 200–220 g were obtained from the animal house of Pharos University in Alexandria (PUA), Alexandria, Egypt. A priori power analysis (G-power analysis software 3.1.9.4) was utilized to calculate the minimum required sample size for an acceptable statistical power) (Cohen, 1988). Animals were housed under standard environmental conditions at 20–22°C and 40–60% humidity in a 12 h light/ 12 h dark cycle with unlimited access to standard rat chow and water.

### Ethical Statement

All experiments were done under minimal distress on rats. The present study applied the guidelines of the "National Research Council's Guide for the Care and Use of Laboratory Animals of the National Institutes of Health" (National Research Council, 2011), the regulations of Egypt's guide for the care and use of laboratory animals (Fahmy and Gaafar, 2016) and the ARRIVE guidelines to minimize distress on rats throughout the experiments.

### Induction of AD-like Cognitive Impairment Model

The AD-like cognitive impairment model was induced by an intracerebroventricular injection of streptozotocin (ICV-STZ) (Sigma Aldrich, St. Louis, MO, USA) (Samy et al., 2016; Poddar et al., 2020). Briefly, the animals were anesthetized with ketamine (90 mg/kg, i.p.) and xylazine (10 mg/kg, i.p.) and were placed into the stereotactic apparatus (Kopf, Germany), and the skulls were exposed. Rats were placed according to the stereotaxic coordinates of the rat brain (0.8 mm posterior to the bregma, 1.8 mm lateral to the sagittal suture, and 3.6 mm beneath the cortical surface). The lateral ventricle on right and left sides were properly marked and a hole was made using a motorized drill and a 20-gauge guide cannula was fixed to the skull (Dubey et al., 2018b). Five µl of the freshly prepared STZ in citrate buffer (pH 4.5) was bilaterally ICV injected at a total dose of 3 mg/kg; 2.5 µl each ventricle (The dose was customized as per each rat’s body weight), at a rate of (1 µl/min) with the help of an injector cannula and a Hamilton syringe. The cannula was kept in place for 2–3 min after micro-infusion to prevent backflow of the injected fluid. The control rats (n = 8) were similarly injected with citrate buffer. After that, rats were injected for 5 days with gentamicin (5 mg/kg, i.p.) and meloxicam (1 mg/kg, S.C.) to prevent sepsis and produce analgesia, respectively (Samy et al., 2016). The mortality rate in ICV-STZ injected rats was 15% within the first two weeks (Details regarding the animal deaths are provided in Supplementary Table 1). Mortality was reported to vary from 10% (Nabavi Zadeh et al., 2023) to 33.33% (Roy et al., 2022). After 14 days of STZ injection, the Morris water maze (MWM) test was carried out to confirm the cognitive impairment and the induction of AD in rats (Samy et al., 2016), Fig. 1.

**Fig. 1:**
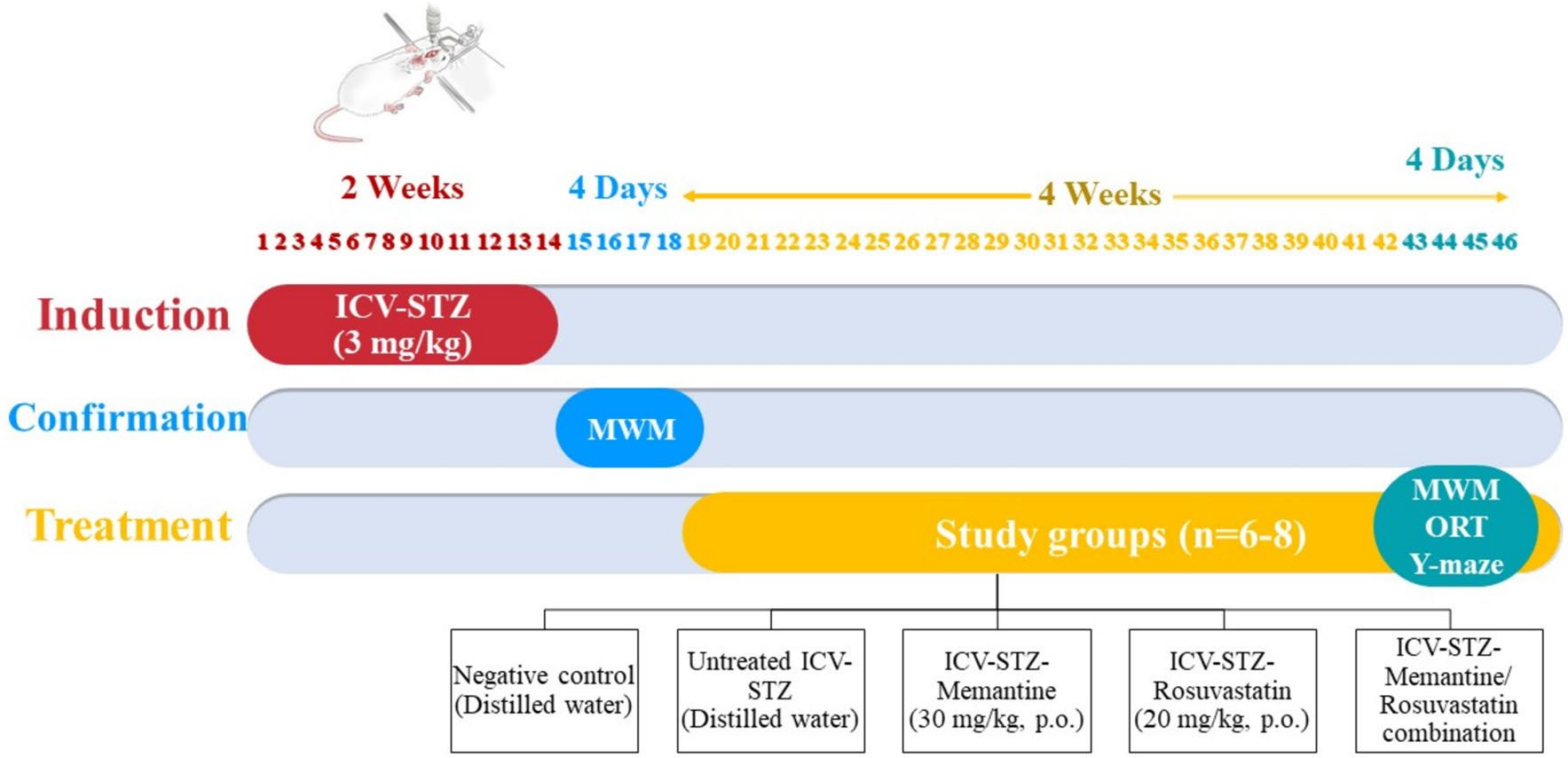
Schematic diagram for the study timeline. (ICV-STZ: Intracerebroventricularly injection of freshly prepared streptozotocin, MWM: Morris water maze, ORT: The object recognition test)

### Experimental Design

Rats were randomly divided into five groups: (*n* = 6–8). Group I (control) served as the negative control group and received distilled water only. ICV-STZ rats were subdivided into 4 groups: Group II (ICV-STZ) served as the untreated ICV-STZ group and received distilled water only; Group III (ICV-STZ-Mem) was treated with memantine (Marcryl, Cairo, Egypt) at a dose of 30 mg/kg/day (Alley et al., 2010); Group IV (ICV-STZ-Ros) was treated with rosuvastatin (Borg Pharmaceutical Industries, Alexandria, Egypt) at a dose of 20 mg/kg/day (Georgieva-Kotetarova and Kostadinova, 2013); Group V (ICV-STZ-Mem-Ros) was treated with a combination both drugs at the aforementioned doses. Treatments with memantine and/or rosuvastatin were gavaged daily for 4 weeks.

During the last 4 days of treatment, animals were subjected to behavioral tests (MWM, object recognition test (ORT), and spontaneous alternation behavior (SAB) in the Y-maze test). Rats were then sacrificed, and hippocampal tissues were immediately excised, divided into aliquots, and preserved at −80°C. According to the manufacturer’s instructions, one aliquot of 30 mg weight was used for DNA and total RNA extraction using DNeasy kit and RNeasy kit (Qiagen, Hilden, Germany), respectively for the determination of p21, ABCA1, LRP1, RAGE, miR-26, miR-185, and miR-205. The second aliquot was used for the determination of Aβ_1−42_, TGF-β1, and p-Smad2 expression by Western Blot technique using V3 Western Workflow™ Complete System, Bio-Rad® (Hercules, CA, USA).

Furthermore, an aliquot of 100 mg of the hippocampal tissues was homogenized in phosphate-buffered saline (PBS) in a ratio of 1:9 and centrifuged. The supernatant homogenate was used for the colorimetric determination of malondialdehyde (MDA), and reduced glutathione (GSH) using commercially available kits (BioVision Incorporated, Milpitas, USA Catalog no. K739 and K261, respectively). The Lowry method was also used to determine protein concentrations (Lowry et al., 1951). Finally, one hemisphere was fixed in 10% formal saline for the histopathological examination.

### Behavioral Assessment

#### Morris Water Maze (MWM)

Each animal was subjected to a spatial reference learning trial in the MWM after ICV-STZ induction and at the end of the treatment period. The MWM is made up of a dark circular pool, 99 cm in diameter, and 56 cm in height. The pool was filled with water at room temperature to a depth of approximately 20 cm, which was made opaque by powdered starch. During the training trail, a submerged circular platform (10 cm diameter), which is the only escape, was positioned in a fixed location in the target quadrant (northeast quadrant) of the maze and was 20 cm away from the edge and 1 cm beneath the water surface. All rats were trained for 3 consecutive days and each training session is made up of four trials. During each trial, every rat was allowed to freely swim for 120 seconds to find the hidden platform. After which, the rat was allowed to stay there for 20 seconds, and was then returned to its holding cage for 30 seconds until the beginning of the next trial. The trial was repeated for each animal in different quadrants. After training, the rats were kept in their home cage. Noting that, if any rat could not find the platform within 120 seconds, the examiner placed it on the plate for 20 sec. Escape latency, which is the total time required to reach the submerged platform, was recorded by a stopwatch. Spatial learning is assessed across repeated trials. In normal rats, the latency to reach the hidden plate form is decreased throughout the training days (Ayieng'a et al., 2023; Shehata et al., 2023). A probe trial, consisting of 60 seconds of free-swimming after removing the hidden platform from the pool, was done on the fourth day. The better the reference memory was consolidated, the greater number of crossovers and the more time the rat swam around the correct location of the removed platform in the target quadrant were observed. Finally, the number of crossovers was counted and the percentage of the time spent in the target quadrant was calculated.

#### The Object Recognition Test (ORT)

The experiments were performed in a wooden box measuring (50 cm × 25 cm × 50 cm). The task procedure includes habituation, familiarization, and test phases. In the habituation phase, animals were habituated for two consecutive days for 10 minutes to the test box with no objects present. In the familiarization phase, animals were kept for 5 minutes in the box as a habituation period. Then after, two similar objects were placed in two corners, approximately 30 cm apart from each other. The time required for each animal to explore each object was recorded for the next 5 minutes (training session). The rats were considered exploring the objects by sniffing, facing, or biting any of the objects. The animals were then returned to their cages (Karasawa et al., 2008). After 120 minutes, the animals were once again positioned in the empty test box as a habituation period, and after 5 minutes, a familiar object and a novel object were directly introduced into the test box. The time required for each animal to explore each object was recorded throughout the next 5 minutes (the test phase).

Finally, discrimination and recognition indices were calculated. Discrimination index (DI) = $\:\frac{(T\:\text{N}\:-\:T\:\text{F})}{(T\:\text{N}\:+\:T\:\text{F})}$, whereas *T*N is the exploration time of the novel object and *T* F is the exploration time of the familiar object (Antunes and Biala, 2012). A positive result means that the rat spent more time exploring the novel object, while a negative score means that the rat spent more time exploring the familiar object, and a zero score indicates a null preference for exploring the two objects (Aggleton et al., 2010). Recognition index (RI), is defined as the time spent exploring the novel object with respect to the total time of both objects exploration [RI = *T*N/(*T*N + *T* F)] (Botton et al., 2010).

#### Spontaneous Alternation Behavior (SAB) in the Y Maze Test

The Y-shaped maze consisted of three arms (A, B & C) at a 120° angle from each other. Each arm was 30 cm long and 8 cm wide with 12 cm height. In the beginning, rats were placed at the center of the Y maze. The sequence of arm entries over a 5-minute period was recorded manually for each rat. Valid entry was recorded when the rat entered with the 4 paws inside the arm. Spontaneous alternation was counted if the rat entered three dissimilar arms consecutively i.e., ABC, CAB, or BCA but not ABB. The Y maze was cleaned with 10% ethanol after each animal to remove odors and any residues. The percent of spontaneous alternation behavior (% SAB) is calculated as the ratio of the actual number of alternations to the possible number of alternations as follows: % SAB = $\:\frac{\text{N}\text{u}\text{m}\text{b}\text{e}\text{r}\:\text{o}\text{f}\:\text{a}\text{l}\text{t}\text{e}\text{r}\text{n}\text{a}\text{t}\text{i}\text{o}\text{n}\text{s}}{\text{T}\text{o}\text{t}\text{a}\text{l}\:\text{a}\text{r}\text{m}\:\text{e}\text{n}\text{t}\text{r}\text{i}\text{e}\text{s}-2}\times\:100$% (Ghafouri et al., 2016). As the percentage of spontaneous alternation increases, as the level of memory retention is higher as normal rats tended to enter the arm that had not been recently entered (El-Mezayen et al., 2022).

### Assessment of miRNAs and p21, ABCA1, LRP1, and RAGE Genes at the mRNA Level

Total RNA, including miRNAs, was isolated from samples using a Trizol RNA isolation kit (Zymo Research Corp., USA, Cat#R2072) according to the supplier’s instructions. RNA quantity and quality were assessed using a Beckman dual spectrophotometer (Thermo Fisher, Massachusetts, USA). MiScript II RT Kit was used (Qiagen, Hilden, Germany, Catalog no. 218161) for the reverse transcription of the extracted RNA, which adopts a one-step, single-tube reverse transcription reaction. Briefly, miScript HiFlex buffer was added to convert mature miRNA, precursor miRNA, non-coding RNA, and mRNA into cDNA, which allows the investigation of miRNA biogenesis and mRNA regulation.

Real-time PCR quantification of mature miRNAs and different assessed genes was performed using the obtained cDNAs by Rotor-Gene Q qPCR (Qiagen, USA) using QuantiTect SYBR Green PCR kit (Qiagen, Hilden, Germany, Catalog no. 204143). The PCR thermal cycling conditions for miRNA were 10 min at 95°C and 40 amplification cycles of 95°C for 2 seconds, 60°C for 20 seconds, and 70°C for 10 seconds. For mRNA, the PCR conditions were 2 min at 50°C, 30 seconds at 95°C, and 40 amplification cycles of 95°C for 5 seconds, and 60°C for 34 seconds. Data collection was done using Rotor-Gene Q-Pure Detection version 2.1.0 (build 9) (Qiagen, USA). The expression of miRNA/mRNA was normalized to the expression of U6/β-actin and quantified using the 2^−ΔΔCt^ method. The relative change in miRNAs or mRNA expression in samples was estimated by normalizing values of their threshold cycles (C_t_) to that of U6 or β-actin, respectively using the ΔΔC_t_ method (Livak and Schmittgen, 2001; El-Mezayen et al., 2022). The primers used for the determination of the assayed genes are listed in Table 1.

**Table 1:** Used oligonucleotide primers in the present study for RT-PCR

Primer		Sequence	Accession number
p21	Forward	5'- GACATCTCAGGGCCGAAA − 3'	NM_080782.4
	Reverse	5'- GGCGCTTGGAGTGATAGAAA − 3'	
ABCA1	Forward	5'- GGTGGTGTTCTTCCTCGTTAC-3'	NM_178095.3
	Reverse	5'- TCCTCGTCCTCGTCATTCAA-3'	
LRP1	Forward	5'- CCACTATGGATGCCCCTAAAAC-3'	NM_001130490.1
	Reverse	5'- GCAATCTCTTTCACCGTCACA − 3'	
RAGE	Forward	5'- CTGCCTCTGAACTCACAGCCAATG − 3'	NM_053336.2
	Reverse	5'- TCCTGGTCTCCTCCTTCACAACTG-3'	
β-actin	Forward	5'- CGGTCAGGTCATCACTATCG-3'	NM_031144.3
	Reverse	5'- TTCCATACCCAGGAAGGAAG-3'	
miRNA-26	Forward	5’- TCAAGTAATCCAGGATAGG-3’	NR_031830.1
	Reverse	5’- GAACATGTCTGCGTATCTC-3’	
miRNA-185	Forward	5’- GGATTGGAGAGAAAGGCAGTTCC-3’	NR_031903.1
	Reverse	5’- GGGAGAGAAGGACCAGAGGAAAG-3’	
miRNA-205	Forward	5’- AATCCATGGGTCCTCCTGTCC-3’	NR_031920.1
	Reverse	5’- TCACTCCACTGAAATCTGGTTGG-3’	
U6	Forward	5'- CTCGCTTCGGCAGCACA-3'	XR_005498700.1
	Reverse	5'- ACGCTTCACGAATTTGCGT-3'	

### Assessment of hippocampal β-Amyloid_1−42_, TGF-β1 and p-Smad2 Protein Expression Level Using Western Blot Technique

Extraction of proteins from tissue was done using ice-cold radioimmunoprecipitation assay (RIPA) buffer with protease and phosphatase inhibitors (50 mmol/L sodium vanadate, 2 mg/mL aprotinin, 0.5 mM phenylmethylsulphonyl fluoride, and 0.5 mg/mL leupeptin), by centrifugation at 12,000 rpm for 20 minutes. Lowry’s method was used to determine the protein concentration for each sample. Separation of equal amounts of protein (20–30 µg of total protein) was achieved by SDS/polyacrylamide gel electrophoresis (10% acrylamide gel) using a Bio-Rad Mini-Protein II system (Bio-Rad Laboratories, USA). A Bio-Rad Trans-Blot System was used to transfer the protein to polyvinylidene difluoride membranes (Pierce, Rockford, IL, USA). Afterward, the membranes were washed and blocked with 3% bovine serum albumin (BSA) in tris-buffered saline with Tween 20 (TBST), which contained 20 mM Tris pH 7.5, 150 mM NaCl, 0.1% Tween 20 for 1 hour at room temperature. Following blocking, the protein blots were developed using antibodies for β-Amyloid_1−42_ (Bioss Antibodies, MA, USA, Catalog no. bs-0107R, 1:1000), as well as TGF β−1 (Catalog no. PA1-9574, 1:2000), p-Smad2 (Catalog no. PA5-37634, 1:1000) and β-actin (Catalog no. PA1-183, 1:1000) (Thermo Fisher Scientific, IL, USA). According to the manufacturer’s instructions, the antibodies were incubated overnight at pH 7.6 at 4°C with gentle shaking. Next, peroxidase-labeled secondary antibodies were added after washing, and then membranes were incubated at 37°C for 1h. ChemiDoc™ imaging system with Image Lab™ software version 5.1 (Bio-Rad Laboratories Inc., Hercules, CA, USA) was used to analyze the band intensity of the target proteins against the control sample after normalization by β-actin protein expression, and the results were expressed as arbitrary units (El-Sayyad et al., 2021).

### Histopathological Assessment

Hippocampal tissues from each group (*n* = 6–8) were sectioned at 5 µm after fixation in 10% formalin and stained with Harris hematoxylin and eosin stain (H&E). One slide with two sections was prepared for each rat. Histomorphometric quantitative analysis using ImageJ software (Image J 1.47v, National Institute of Health, Bethesda, MD, USA) was performed to quantify the number of normal neurons, degenerated neurons, and glial cells. All the histopathological examinations and measurements were done in a blinded manner.

### Statistical Analysis

Values are expressed as means ± S.E.M, (n = 6–8) and analyzed using GraphPad Prism v 7.0 (La Jolla, CA, USA). The behavioral data in MWM was analyzed by two-way repeated measures ANOVA followed by Bonferroni’s post hoc test. Unpaired t-test was also used to compare between the normal and the ICV-STZ rats before treatment. In addition, one-way ANOVA followed by Tukey’s post hoc test was performed for the comparison of the probe trial test findings of MWM test and other different parameters in all studied groups. The correlation coefficients (r) between different parameters were defined using the Pearson correlation coefficient; *P ≤* 0.05 was the limit of significance for all the studied comparisons. A factorial design test was used to determine the potential interaction between individual treatments when given in combination.

## Results

### Behavioral Tests

#### Examination of Cognitive and Learning Abilities Using Morris Water Maze (MWM)

**Fig. 2:**
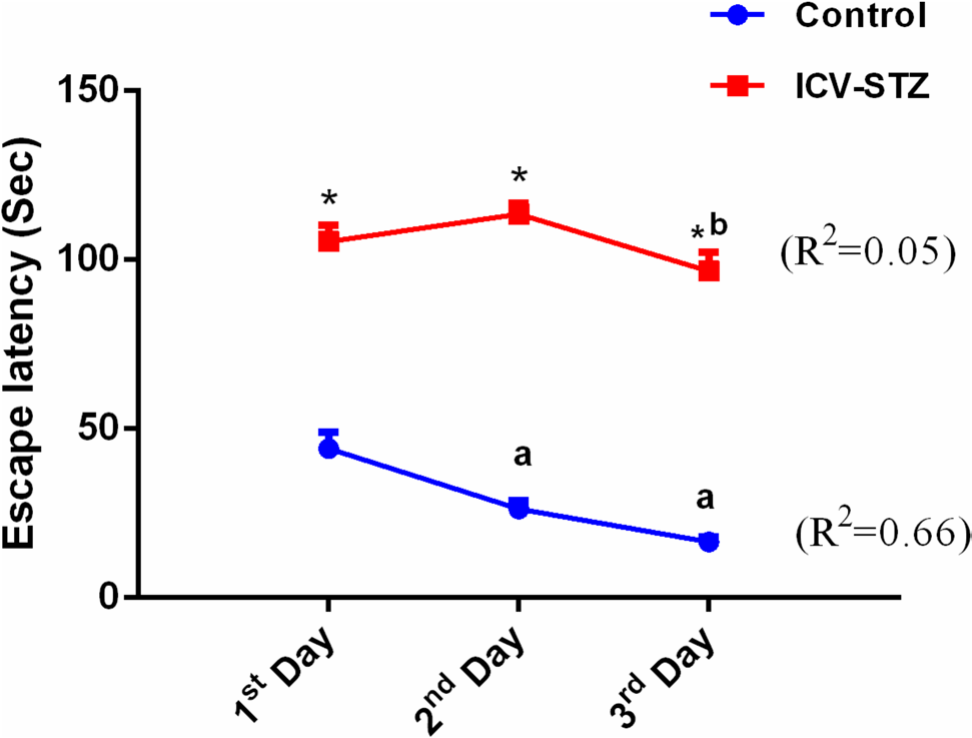
Effect of ICV-STZ (3 mg/kg) administration on the mean escape latency (sec) in the MWM test in male rats. Data are presented as mean ± S.E.M (n = 8 control, n = 26 ICV-STZ) and were analyzed statistically using two-way repeated measures ANOVA followed by Bonferroni’s post hoc test (*P* < 0.05) as compared with the corresponding value of control (*), 1^st^ day within the same group (**a**), 2^nd^ day within the same group (**b**). Values in parentheses indicate the correlation coefficient using linear regression analysis for control and ICV-STZ-treated rats

As illustrated in Table 2 and Fig. 2, the findings showed a marked increase in the escape latency of the ICV-STZ rats on day 3rd (Column Factor F_(1,32)_ = 254.6, *P* < 0.0001, Time F_(2,64)_ = 2.052, NS, Interaction F_(2,64)_ = 1.962, NS), besides significant decreases in the percentage time spent in the target quadrant (t = 6.7195, df = 32) and number of crossovers (t = 11.965, df = 32) when ICV-STZ rats were compared to normal rats, confirming the impairment of learning parameters and the development of AD model.

**Table 2:** Effect of intracerebroventricular injection of streptozotocin (3 mg/kg) (ICV-STZ) on Morris water maze test in male rats

Group	Escape latency (Sec)			% time spent in the target quadrant	Number of crossovers
	1^st^ Day	2^nd^ Day	3^rd^ Day		
Control	39.9 ± 4.6	28.6 ± 2.2^a^	16.5 ± 1.7^a^	39.6 ± 2.7	3.8 ± 0.34
ICV-STZ	110.6 ± 5.0^*^	113.5 ± 4.4^*^	110.4 ± 4.8^*b^	23.0 ± 1.1^*^	0.87 ± 0.09^*^

Data are presented as mean ± S.E.M (*n* = 8 control, 26 ICV-STZ) and were analyzed statistically using two-way repeated measures followed by Bonferroni’s *post hoc* test (*P* < 0.05) and unpaired t-test as compared with corresponding value of control (^*^), 1^st^ day within the same group (^a^), 2^nd^ day within the same group (^b^)

**Fig. 3:**
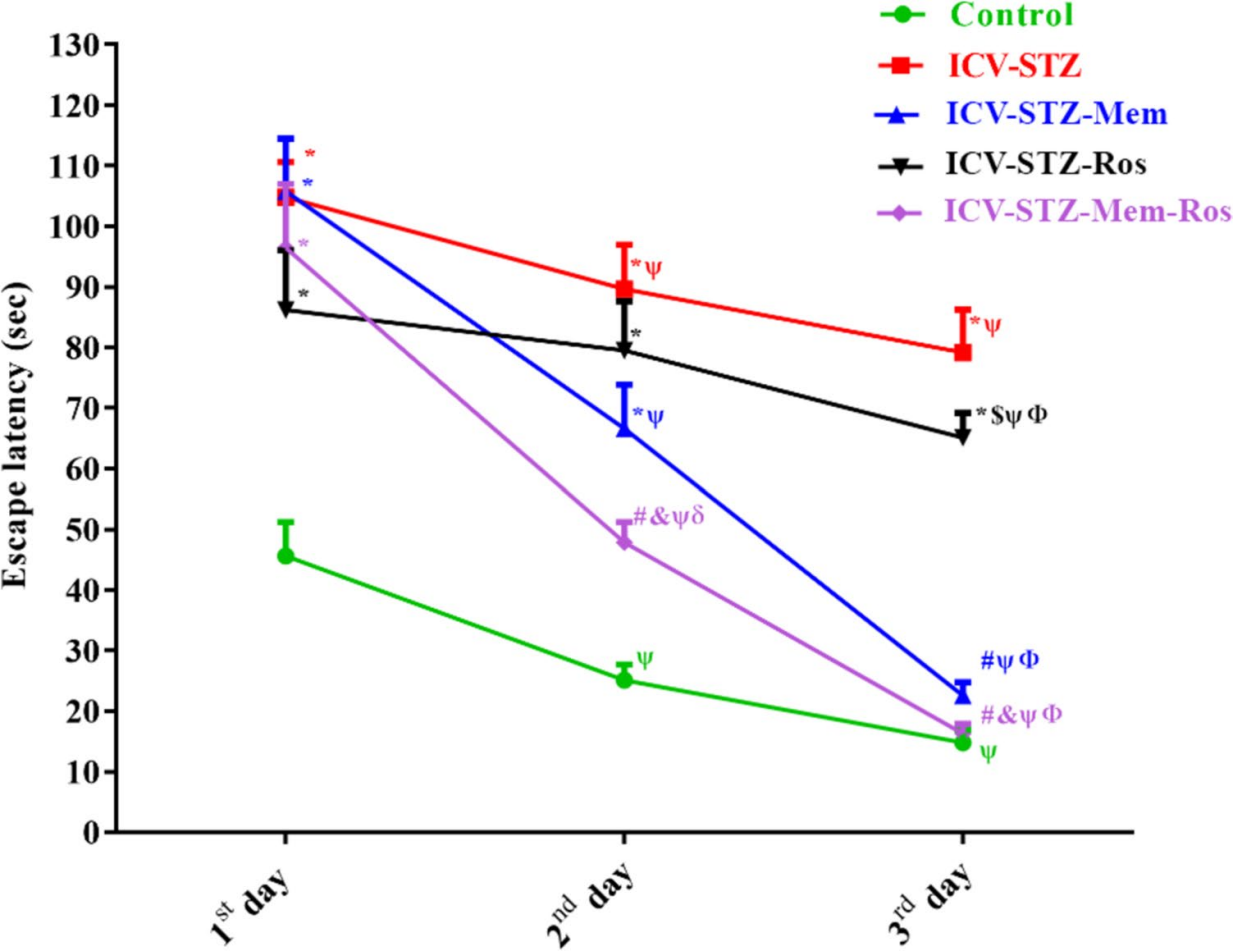
Effect of memantine (30 mg/kg/day), rosuvastatin (20 mg/kg/day), and their combination on the escape latency during acquisition in the ICV-STZ treated rats. Memantine (30 mg/kg/day), rosuvastatin (20 mg/kg/day) and their combination were gavaged orally for 28 days after AD model establishment. Data are presented as mean ± S.E.M (n = 6–8) and were analyzed statistically using two-way repeated measures followed by Bonferroni’s post hoc test (*P* < 0.05). As compared with (*) control, (#) ICV-STZ, ( $ ) ICV-STZ-memantine, (&) ICV-STZ-rosuvastatin-treated groups; (Ψ and Φ) as compared to day 1 and day 2, respectively, (δ) indicate potentiating interaction using Factorial Design Drug interaction test

As presented in Fig. 3, the ICV-STZ group showed a significantly longer time to reach the submerged platform (latency) with respect to the control group (Column Factor F_(4, 29)_ = 56.31, *P* < 0.0001, Time F_(2,58)_ = 87.88, *P* < 0.0001, Interaction F_(8,58)_ = 6.975, *P* < 0.0001). Different treatments altered the learning ability of the rats by changing the latency during the 3 days of training trials. This effect was confirmed by a significant decrease in the mean latencies to reach the hidden platform during acquisition in memantine and its combination with rosuvastatin as compared to the untreated ICV-STZ group, showing a potentiating interaction on the 2^nd^ day. Both groups showed a non-significant difference when compared to the normal rats on the third day. While in the probe trial (the 4^th^ day), ICV-STZ rats showed a significant reduction in the percentage of time spent in the target quadrant, number of crossovers and swimming speed as compared to the normal control rats as shown in Table 3. In contrast, memantine alone and memantine/rosuvastatin combination showed a significant reduction in the escape latency, reaching 27.46% and 19.81% of the AD value in the third day, respectively; values that were comparable to normal values. In addition, these groups showed a significant increase in the percentage of time spent in the target quadrant to 1.85 and 1.66 folds, respectively (Treatment F_(4, 29)_ = 37.285, *P* < 0.0001), as well as the number of crossovers to 7.71 and 7.86 folds, respectively (Treatment F_(4, 29)_ = 22.395.72, *P* < 0.0001) and the swimming speed to 4.18 and 4.5 folds as compared to the ICV-STZ-rats (Treatment F_(4, 29)_ = 30.10051, *P* < 0.0001), indicating improvements in learning and cognitive performance. On the other hand, rosuvastatin alone failed to show any significant effects as compared to the ICV-STZ group, except for the swimming speed.

**Table 3:** Effect of memantine (30 mg/kg/day), rosuvastatin (20 mg/kg/day), and their combination on Morris water maze, object recognition, and Y-Maze spontaneous alternation behavior tests in ICV-STZ rats

	Morris water maze (MWM)			Object recognition test (ORT)		Spontaneous alternation behavior (SAB)
Groups	% time in target quadrant	Number of crossovers	Swimming speed (cm/s)	Discrimination index	Recognition Index	% SAB
Control	46 ± 1.8	5.6 ± 0.54	16.2 ± 0.44	0.76 ± 0.08	0.88 ± 0.04	70.4 ± 1.17
ICV-STZ	24.4 ± 1.4^*^	0.7 ± 0.36^*^	3.8 ± 0.28^*^	0.02 ± 0.1^*^	0.51 ± 0.05^*^	44.8 ± 1.9^*^
ICV-STZ-Memantine (30 mg/kg/day)	45.2 ± 1.4^#^	5.4 ± 0.61^#^	15.9 ± 0.48^#^	0.50 ± 0.07^#^	0.74 ± 0.04^#^	73.6 ± 6.4^#^
ICV-STZ-Rosuvastatin (20 mg/kg/day)	24.6 ± 1.9^*$^	1.6 ± 0.46^*$^	8.7 ± 0.36^*#$^	0.24 ± 0.07^*^	0.62 ± 0.03^*^	51.0 ± 1.8^*$^
ICV-STZ-Memantine/ Rosuvastatin	40.6 ± 2.1^#&^	5.5 ± 0.53^#&^	17.1 ± 0.44^#&^	0.74 ± 0.09^#& δ^	0.87 ± 0.05^#&δ^	68.4 ± 2.7^#&^

Memantine (30 mg/kg/day), rosuvastatin (20 mg/kg/day) and their combination were gavaged orally for 28 days after AD model establishment. Data are presented as mean ± S.E.M (*n* = 6–8) and were analyzed statistically using one-way ANOVA followed by Tukey’s *post hoc* test (*P* < 0.05). As compared with (^*^) control, (^#^) ICV-STZ, ($) ICV-STZ-memantine, (^&^) ICV-STZ-rosuvastatin-treated groups; (δ) indicates potentiating interaction using Factorial Design Drug interaction test.

#### Examination of Cognitive Ability Using the Object Recognition Test (ORT)

As presented in Table 3, the ICV-STZ group showed a significant reduction in the discrimination and recognition indices to 2.63% and 57.95% of the normal values with an impairment in the exploratory behavior and novel object recognition as well as a reduction in the differentiation ability based on attention. Nevertheless, there was a significant increase in both indices in the memantine and the combination-treated groups as compared to the untreated ICV-STZ group; effects that were comparable to the normal values. Again, rosuvastatin alone failed to show any significant reduction in discrimination (Treatment F_(4, 29)_ = 15.001, *P* < 0.0001), and recognition indices (Treatment F_(4, 29)_ = 14.112, *P* < 0.0001). In addition, the combination showed a potentiating interaction in both parameters.

### Influence of Memantine, Rosuvastatin and their Combination on Hippocampal Amyloid β_1−42_(Aβ_1−42_), Transforming Growth Factor β1 (TGF-β1), Phospho-Smad2 (p-Smad2) and p21 Gene

**Fig. 4:**
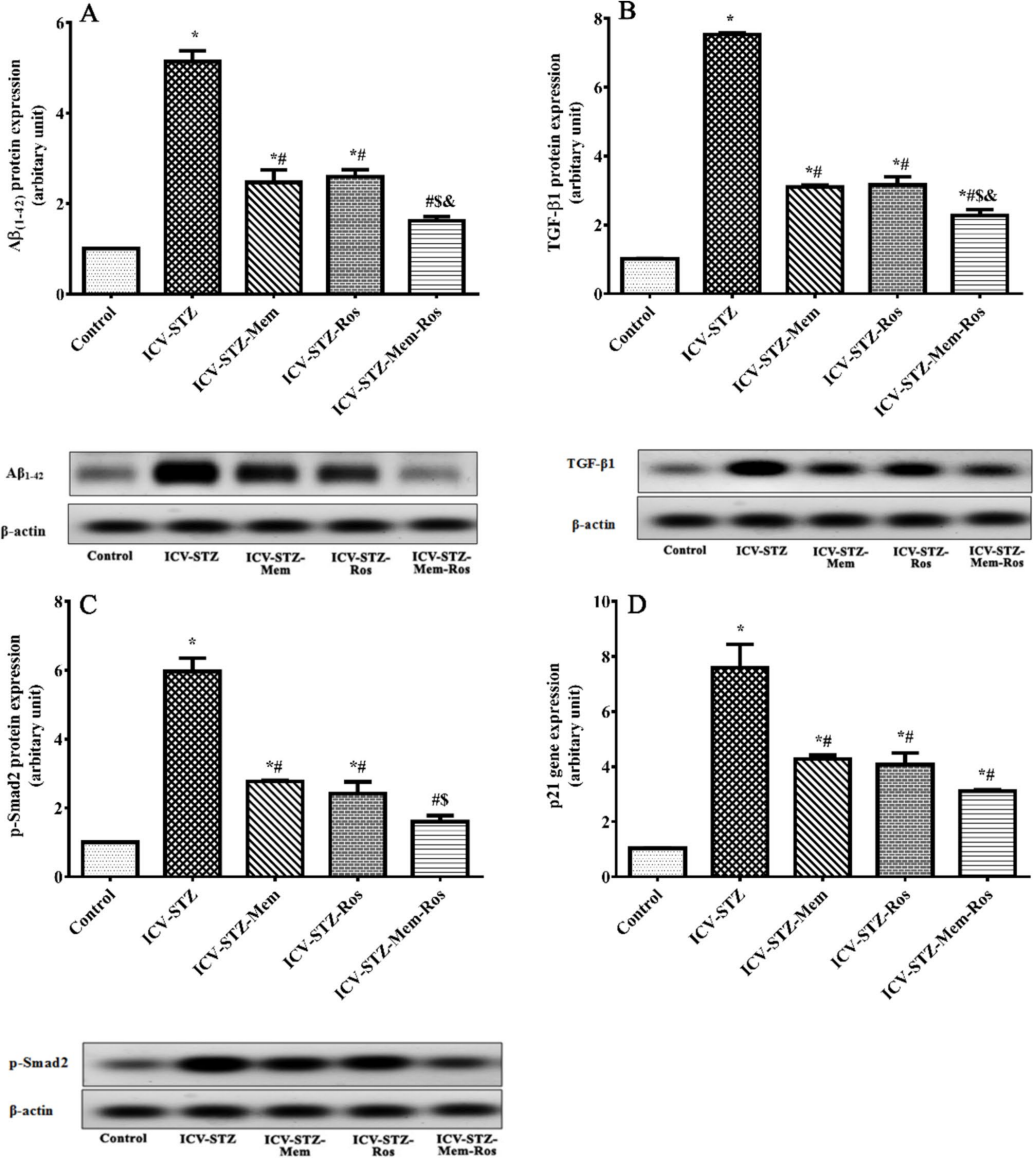
Effect of memantine (30 mg/kg/day), rosuvastatin (20 mg/kg/day), and their combination on amyloid β, TGF-β1/Smad2/p21 in the hippocampus of ICV-STZ rats. (**A**) Aβ 1−42 , (**B**) TGF-β1, (**C**) p-Smad2, and (**D**) p21 gene. Results were normalized to β-actin as a reference gene. Data are presented as means of 6–8 rats ± S.E.M and were analyzed statistically using one-way ANOVA followed by Tukey’s post hoc test (*P* < 0.05). As compared with (*) control, (#) ICV-STZ, ( $ ) ICV-STZ-memantine, (&) ICV-STZ-rosuvastatin-treated groups. Memantine (30 mg/kg/day), rosuvastatin (20 mg/kg/day) and their combination were gavaged orally for 28 days after model establishment. (Aβ 1−42 : amyloid β 1−42 ; TGF-β1: transforming growth factor β1; p-Smad2: phospho-Smad2)

ICV-STZ injection significantly increased the hippocampal content of Aβ_1−42_ by 5-folds compared to normal control group (Treatment F_(4, 29)_ = 185.992, *P* < 0.0001). However, treatment with memantine, rosuvastatin and their combination significantly decreased Aβ expression level to about 48%, 50% and 31%, respectively, compared to the ICV-STZ rats as shown in Fig. 4A. Regarding TGF-β1/Smad pathway, ICV-STZ rats showed a significant elevation in the TGF-β1, p-Smad2 and p21 expression level in the hippocampus by 650%, 497% and 660%, respectively, in relation to the control group. On contrast, treatment with memantine, rosuvastatin and their combination showed a significant reduction in the TGF-β1, p-Smad2 and p21 expression level in the hippocampus to various extents when compared the ICV-STZ rats as shown in Fig. 4 (B-D). Memantine and rosuvastatin treatment showed comparable results on all the aforementioned parameters. Notably, the combination treatment normalized Aβ_1−42_, p-Smad2 and p21 levels, showing the best ameliorating effect.

### Influence of Memantine, Rosuvastatin and their Combination on BBB Transporters and their Regulating miRNAs

**Fig. 5:**
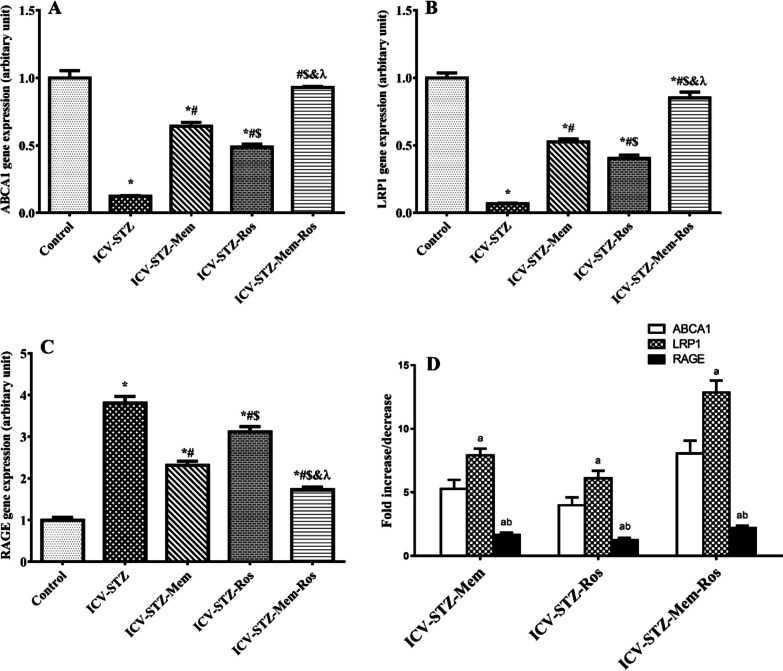
Effect of memantine (30 mg/kg/day), rosuvastatin (20 mg/kg/day), and their combination on gene expression of BBB transporters in rats with ICV-STZ-induced AD. (A-C): Comparison between different groups according to mRNA expression of (**A**) ABCA1, (**B**) LRP1 and (**C**) RAGE. (**D**): Comparison between the effects of each drug on different BBB transporters. Results were normalized to β-actin as a reference gene. Data are presented as mean ± S.E.M (*n* = 6–8) and were analyzed statistically using one-way ANOVA followed by Tukey’s post hoc test (*P* < 0.05). As compared with (*) control, (#) ICV-STZ, ($) ICV-STZ-memantine, (&) ICV-STZ-rosuvastatin-treated groups; a: Statistically significant compared to ABCA1 expression, b: Statistically significant compared to LRP1 expression. λ: indicates additive interaction using Factorial Design Drug interaction test. Memantine (30 mg/kg/day), rosuvastatin (20 mg/kg/day) and their combination were gavaged orally for 28 days after model establishment. (ABCA1: ATP-binding cassette transporter A1, LRP1: LDL receptor–related protein 1, RAGE: receptor for advanced glycation end products)

**Fig. 6:**
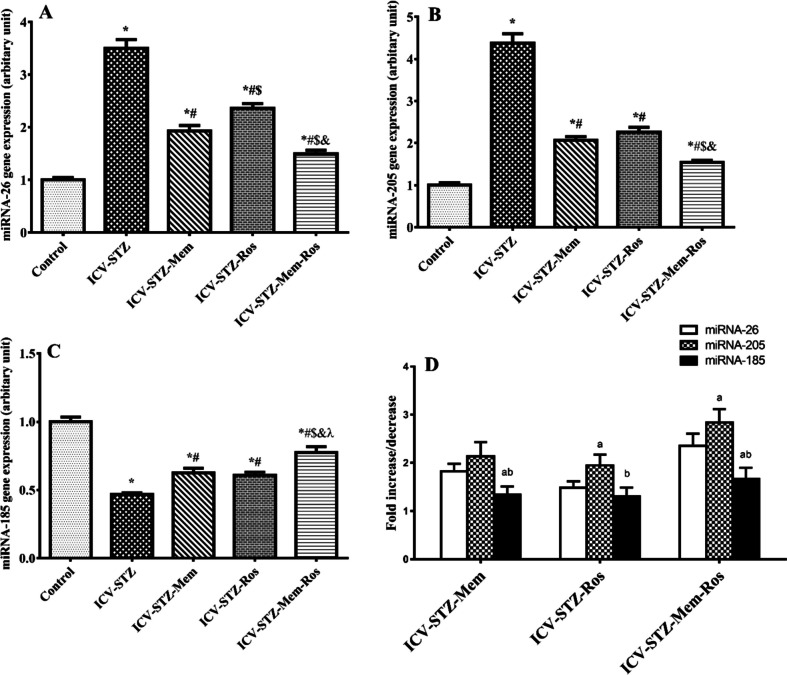
Effect of memantine (30 mg/kg/day), rosuvastatin (20 mg/kg/day), and their combination on the expression of miRNAs regulating BBB transporters in rats with ICV-STZ-induced AD. **A**-**C**: Comparison between different groups according to the expression of (**A**) miRNA-26, (**B**) miRNA-205 and (**C**) miRNA-185. (**D**): Comparison between the effects of each drug on different miRNAs. Results were normalized to U6 as a reference gene. Data are presented as means of 6–8 rats ± S.E.M and were analyzed statistically using one-way ANOVA followed by Tukey’s post hoc test (P < 0.05). As compared with (*) control, (#) ICV-STZ, ($) ICV-STZ-memantine, (&) ICV-STZ-rosuvastatin-treated groups; a: Statistically significant compared to 26 expression, b: Statistically significant compared to 205 expression. Memantine (30 mg/kg/day), rosuvastatin (20 mg/kg/day) and their combination were gavaged orally for 28 days after model establishment. (ABCA1: ATP-binding cassette transporter A1, LRP1: LDL receptor–related protein 1, RAGE: receptor for advanced glycation end products)

In the current investigation, three BBB transporters were assessed at their gene level; ABCA1, LRP1 and RAGE. In the untreated ICV-STZ rats, the expression of ABCA1 and LRP1 decreased to 12% (Treatment F_(4, 29)_ = 163.972, *P* < 0.0001), and 6.7% (Treatment F_(4, 29)_ = 195.180, *P* < 0.0001) of the control rats, respectively, whereas the expression of RAGE significantly increased to reach 3.81 folds as compared to the control rats (Treatment F_(4, 29)_ = 126.594, *P* < 0.0001). Memantine and/or rosuvastatin significantly increased the expression of ABCA1 and LRP1 and decreased the expression of RAGE to various extents. Amongst all treated groups, the highest levels of both ABCA1 and LRP1 (7.96 and 12.7 folds) and the lowest RAGE level (45.32%) were observed in the group receiving the combination therapy as compared to the untreated ICV-STZ rats; values that were statistically significant difference when to compared to memantine and rosuvastatin alone treatments, Fig. 5. Upon comparing the effect of each drug on different BBB transporters, it was found that the tested therapeutics more significantly affected LRP1 expression (the greatest number of fold change) than ABCA1 and RAGE expressions. All therapeutics showed their least effect on RAGE expression (the least number of fold change), which was significantly less than their effect on other BBB transporters, Fig. 5D. As illustrated in Fig. 6, the miRNA modulating ABCA1, LRP1 and RAGE expression; miRNA-26, miRNA-205 and miRNA-185, respectively, were analyzed. The untreated ICV-STZ rats showed a statistically significant increase in miRNA-26 and miRNA-205 to 3.5 and 4.38 folds (Treatment F_(4, 29)_ = 111.211, *P* < 0.0001) and (Treatment F_(4, 29)_ = 132.495, *P* < 0.0001)., respectively, as well as a significant reduction in miRNA-185 to 46.59% rats (Treatment F_(4, 29)_ = 51.219, *P* < 0.0001) as compared to control group. Memantine, rosuvastatin and memantine/rosuvastatin-treatments significantly increased miRNA-26 and miRNA-205 and decreased miRNA-185 relative to untreated AD control rats. There was no statistically significant difference between memantine and rosuvastatin sole treatments and their combination regarding miRNA-205 and miRNA-185 expressions. Yet, rosuvastatin showed significantly higher miRNA-26 expression than memantine (*P* < 0.05). On the other hand, the memantine/rosuvastatin combined therapy showed significantly higher miRNA-185 and less miRNA-205 and miRNA-26 compared to either treatment alone, Fig. 6. For comparing the effect of each drug on different tested miRNAs, the number of fold change in miRNA expression relative to untreated positive control values were calculated and compared, Fig. 6D. It was observed that tested therapeutics pursued the greatest effect on miRNA-205 and the least effect was on miRNA-185. For the combination therapy, they exerted the greatest effect on miRNA-205, which was significantly higher than its effect on other mi-RNAs.

### Influence of Memantine, Rosuvastatin and their Combination on Oxidative Stress Markers

#### Reduced Glutathione (GSH) and Malondialdehyde (MDA) Levels in ICV-STZ Rats

**Fig. 7:**
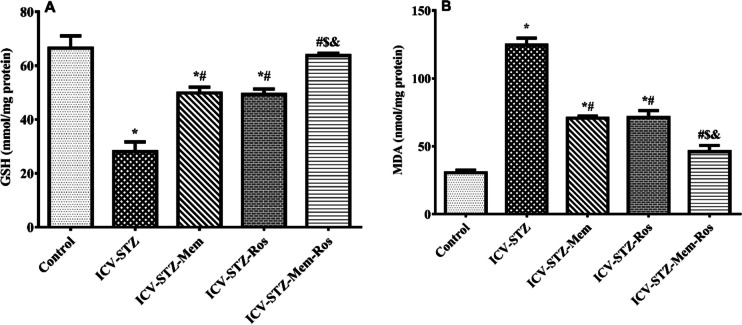
Effect of memantine (30 mg/kg/day), rosuvastatin (20 mg/kg/day), and their combination on oxidative stress in rats with ICV-STZ-induced AD. (**A**) GSH and (**B**) MDA. Data are presented as means of 6–8 rats ± S.E.M and were analyzed statistically using one-way ANOVA followed by Tukey’s post hoc test (*P* < 0.05). As compared with (*) control, (#) ICV-STZ, ($) ICV-STZ-memantine, (&) ICV-STZ-rosuvastatin-treated groups. Memantine (30 mg/kg/day), rosuvastatin (20 mg/kg/day) and their combination were gavaged orally for 28 days after model establishment. (GSH: reduced glutathione; MDA: malondialdehyde)

The ICV-STZ group showed a significant reduction in GSH to about its half and a significant increase (4-folds) in the MDA content as compared to the control group (Fig. 7). On the other hand, GSH content in the rats treated with memantine, rosuvastatin and their combination showed a significant increase by 77%, 79% and 130%, respectively, as compared the ICV-STZ rats (Treatment F_(4, 29)_ = 39.104, *P* < 0.0001). In addition, different treatments showed a significant reduction in the MDA content to 57%, 59% and 36%, respectively, compared to ICV-STZ treated rats (Treatment F_(4, 29)_ = 97.240, *P* < 0.0001). Memantine and rosuvastatin showed comparable effects on oxidative stress parameters. Moreover, the effect of both memantine and rosuvastatin in combination on GSH and MDA was more significant than either drug alone and even normalized their values.

### Histopathological Examination and Morphometric Analyses of Hippocampi from Brain Tissues of Different Studied Groups

**Fig. 8:**
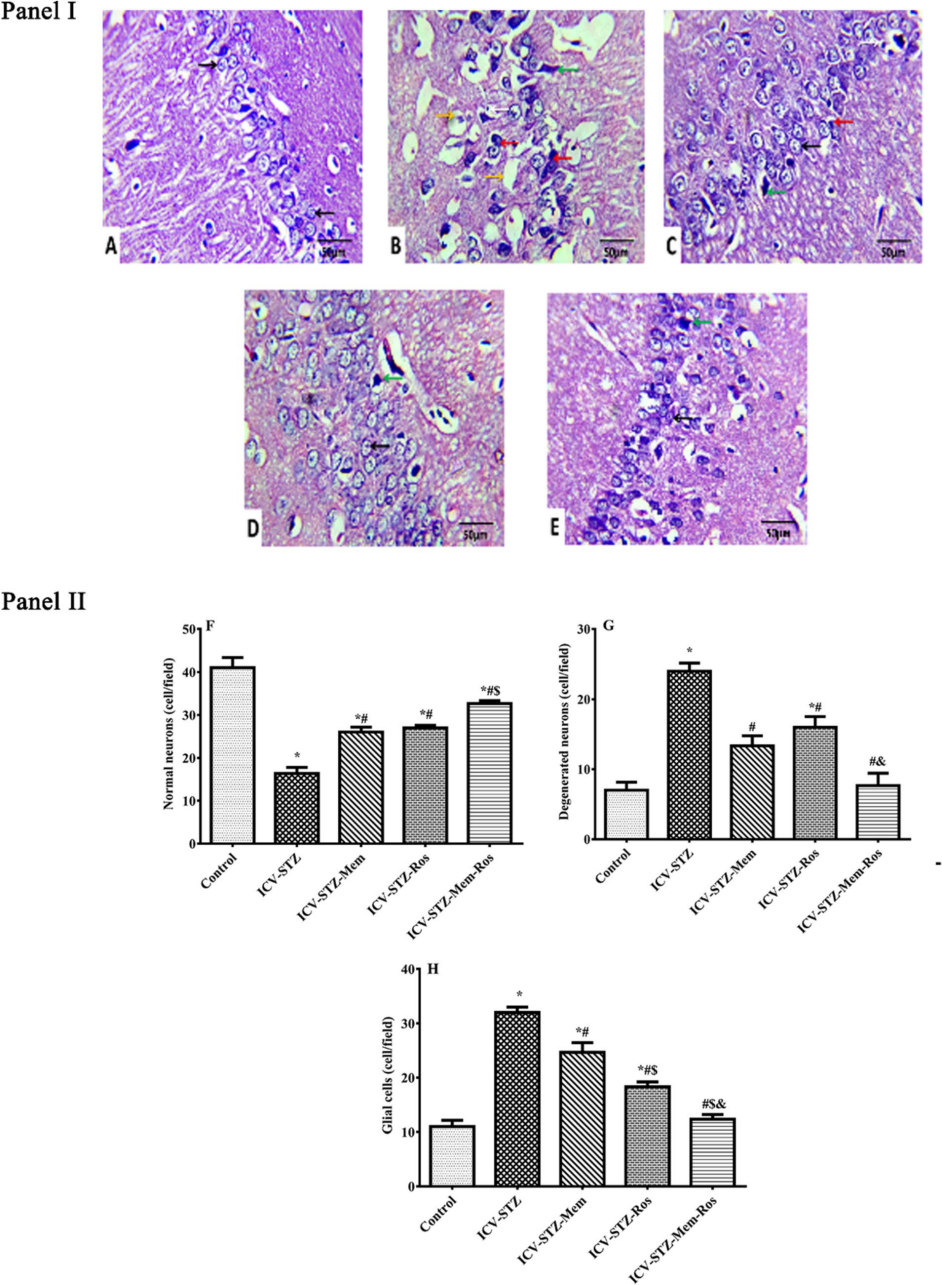
Photomicrographs and morphometric analysis of hippocampus CA1 region stained with hematoxylin and eosin H&E (X400) showing the effect of different treatments on ICV-STZ rats. Panel I : sections from control rats (**A)**, ICV-STZ rats (**B**), ICV-STZ-memantine-treated rats (30 mg/kg/day) (**C**), ICV-STZ-rosuvastatin treated rats (20 mg/kg/day) (**D**) and ICV-STZ-combination-treated rats (**E**). Black arrows: Normal nerve cells, yellow arrows: pericellular space, green arrows: degenerated, pyknotic neurons, white arrows: necrotic neurons associated with satellitosis, and red arrows: necrotic neurons associated with neuronophagia. Panel II shows the hippocampal differential count of normal neurons (**F**), degenerated neurons (**G**), and glial cells (**H**) in the ICV-STZ rats. Data are presented as means of 6–8 measures ± S.E.M and were analyzed statistically using one-way ANOVA followed by Tukey’s post hoc test (*P* < 0.05). As compared with (*) control, (#) ICV-STZ, ($) ICV-STZ-memantine, (&) ICV0-STZ-rosuvastatin-treated groups. Memantine (30 mg/kg/day), rosuvastatin (20 mg/kg/day) and their combination were gavaged orally for 28 days after model establishment

The histopathological examination of brain hippocampus of the different studied groups of male rats is represented in Fig. 8. The control rats showed a normal architecture of the hippocampus, well-organized pyramidal neuronal cell layer with large vesicular nuclei and normal nerve cells (Fig. 8A). ICV-STZ rats showed marked changes in neurons and nerve fibers; the hippocampus showed disorganization of its pyramidal cell layer with neuronal loss. There was also distorted cellular morphology associated with an increased pericellular space, and misaligned, degenerated, shrunken and darkly stained pyknotic neurons necrotic neurons associated with satellitosis and neuronophagia as shown in Fig. 8B. Different treatment regimens showed marked improvements in the overall nerve cell morphology, which were associated with fewer numbers of degenerated, pyknotic and necrotic neurons represented in Fig. 8 (C-E). The best ameliorating effect was observed in the combination treated group.

Regarding the morphometric analysis (Fig. 8 F-H), the count of normal neurons in the ICV-STZ rats was significantly reduced to about 40% that of the control rats. Nevertheless, treatment with memantine or rosuvastatin showed a significant rise in the number of normal neurons by approximately 60% and 66%, respectively, compared to the ICV-STZ rats. Meanwhile, combination of both memantine and rosuvastatin significantly increased the number of normal neurons to double the value of ICV-STZ treated rats; an effect that was significantly prominent than treatment with memantine alone.

In contrast, ICV-STZ untreated rats showed a significant increase in the number of degenerated neurons and glial cells to approximately triple the value of control normal rats. However, treatment with memantine, rosuvastatin and their combination significantly decreased the number of degenerated neurons and glial cells to about 77%, 67%, 32% and to 55%, 57%, 38%, respectively, as compared to ICV-STZ rats. Of note, treatment with the memantine/rosuvastatin combination showed a significant reduction in the number of glial cells as compared to either drug alone and efficiently normalized the number of degenerated neurons and glial cells.

### Correlation Studies

Table 4 shows the correlation between the expression of TGF-ꞵ protein level with behavioral assessments, hippocampal differential cell count, different BBB transporters, and their regulatory miRNA expressions in the positive control and different treated groups. In all tested groups, TGF-ꞵ was found to be highly correlated to ABCA1/miRNA-26 and LRP1/miRNA-205 expressions than RAGE/miRNA-185 expression as confirmed by the *P* value.

**Table 4:** Correlations between TGF-β1 and behavioral assessments, hippocampal differential cell count, the blood brain barrier (BBB) transporters and their regulatory miRNAs in the different studied groups

		TGF-β1				
		Untreated ICV-STZ		ICV-STZ-Memantine	ICV-STZ-Rosuvastatin	ICV-STZ-Memantine/ Rosuvastatin
% time spent in the target quadrant	r (P)	−0.887 (0.018)		−0.934 (0.020)	−0.718 (NS)	−0.920 (0.027)
Number of crossovers	r (P)	−0.936 (0.006)		−0.951 (0.013)	−0.917 (0.028)	−0.969 (0.031)
Discrimination index (DI)	r (P)	−0.940 (0.017)		−0.927 (0.023)	−0.875 (NS)	−0.986 (0.014)
Recognition index (RI)	r (P)	−0.938 (0.018)		−0.917 (0.029)	−0.872 (NS)	−0.992 (0.008)
SAB%	r (P)	−0.832 (0.040)		−0.939 (0.018)	−0.889 (0.044)	−0.941 (0.017)
Normal neurons count	r (P)	−0.847 (0.033)		−0.884 (0.019)	−0.872 (0.023)	−0.839 (0.037)
Degenerated neurons count	r (P)	0.895 (0.16)		0.842 (0.035)	0.879 (0.021)	0.851 (0.031)
Glial cells count	r (P)	0.877 (0.022)		0.822 (0.044)	0.866 (0.026)	0.860 (0.028)
ABCA1	r (P)	−0.952 (0.003)		−0.919 (0.01)	−0.866 (0.026)	−0.914 (0.011)
LRP1	r (P)	−0.902 (0.014)		−0.928 (0.008)	−0.953 (0.003)	−0.853 (0.031)
RAGE	r (P)	0.841 (0.036)		0.874 (0.023)	0.845 (0.034)	0.817 (0.047)
miR-26	r (P)	0.949 (0.004)		0.951 (0.004)	0.853 (0.031)	0.929 (0.007)
miR-205	r (P)	0.921 (0.009)		0.890 (0.017)	0.927 (0.008)	0.910 (0.012)
miR-185	r (P)	−0.891 (0.017)		−0.819 (0.046)	−0.839 (0.037)	−0.886 (0.019)

*r;* Correlation coefficient, *P;* *p*-value, *NS;* non-significant

## Discussion

This study is the first to report the ameliorative effects of memantine and/or rosuvastatin on the cognitive derangements in AD rats that are partly hinged on the modulation of TGF-β1/Smad/p21 signaling and transporters governing the clearance of Aβ across the BBB along with their associated microRNAs. Current novel findings showed that memantine/rosuvastatin combination: i) improved memory retention, spatial acquisition, cognitive performance and short-term spatial working memory in three behavioral tests, namely; Morris water maze, object recognition test and Y maze, ii) improved antioxidant capacity and restored neuronal abnormalities in the CA1 region of the hippocampus, iii) down-regulated TGF-β1/p-Smad2/p21 signaling, and iv) elevated ABCA1/miRNA-26 and LRP1/miRNA-205 expressions associated with Aβ clearance. The interesting firstly reported findings in this study that a strong correlation existed between TGF-ꞵ1 down-regulation with ABCA1/miRNA-26 and LRP1/miRNA-205 expressions and the ability of memantine/rosuvastatin combination to improve cognitive skills and neuronal integrity. Collectively the data presented a novel molecular target of memantine-rosuvastatin combination in the Alzheimer’s model in rats implicating the Aβ clearance to slow down the degenerative processes and memory loss.

Induction of AD using ICV-STZ injection resulted in significant cognitive impairment, oxidative stress, defective Aβ clearance, and histopathological abnormalities that may be attributable to the upsurged TGF-β1/p-Smad2/p21 signaling. Notably, ICV-STZ injection impaired learning and short-term spatial working memory. Moreover, a notable rise in the hippocampal level of Aβ, TGFβ1, p-Smad2 proteins, p21, and MDA as well as a decline in GSH. These findings were histopathologically confirmed by marked gliosis and neuronal loss in the CA1 region of the hippocampus, exhibiting the comprehensive features of cognitive impairment and neuroinflammation in AD rats as shown herein a correlative relationship. These results were in accordance with previous studies (Verma et al., 2020; Tiwari et al., 2021). Our current investigation of CA1 was based on its early involvement in neurodegeneration and atrophy following STZ administration. It was previously reported that the CA1 region of the hippocampus is more likely to be damaged and higher susceptibility to cognitive dysfunction than the DG region in STZ-induced memory deficit model (Agrawal et al., 2011). Indeed, studies have shown the regional specificity of the hippocampus in memory processes. The involvement of CA3 seems to be important at the earliest stage of acquisition, presumably for developing an instant representation of a context. The CA1 and dentate gyrus (DG) were critically involved in retrieving contextual memory after a long time period (Lee and Kesner, 2004; Agrawal et al., 2011), whereas the CA1 region of the hippocampus is involved in cognitive processes, particularly learning, and memory (Zhao et al., 2016); all these characteristics render CA1 region appropriate focus in the current study. ICV-STZ is reported to chronically diminish cerebral glucose uptake and develop insulin resistance in the brain, promoting pathological deposition of insoluble and neurotoxic Aβ_1−42_ and oxidative stress (Grieb, 2016), with the consequential hypersecretion of various cytokines including TGF-β−1 (Olajide and Sarker, 2020). It is worth mentioning that the current investigation was conducted on male Sprague Dawley rats as male, but not female, Sprague Dawley rats are susceptible to ICV-STZ learning and memory impairment (Bao et al., 2017). In addition, female Sprague-Dawley rats display differences in some AD typical biomarkers compared to those observed in humans (Moreira-Silva et al., 2019).

Treatment with memantine alone or combined with rosuvastatin significantly ameliorated the cognitive derangements manifested as improved retention memory, spatial acquisition, cognitive performance, short-term spatial working memory, exploratory behavior, and novel object memory. However, rosuvastatin alone failed to offer a significant improvement in cognition performance despite its added value as a combined therapy. On the molecular level, drug treatments restored the antioxidant capacity, ameliorated hippocampal Aβ, TGF-β1, and p-Smad2 protein levels, amended p21 gene expression, and restored the histopathological insults. The ameliorative effect of combined memantine /rosuvastatin therapy was superior to either drug alone, which could be attributed to the additive effect of both drugs on dampening of the TGF-β1 signaling pathway, which operates a downstream cascade to modulate neuronal integrity.

Memantine protects the brain from calcium-mediated neurodegeneration and glutamate excitotoxicity, endorsing neuronal survival and inhibiting Aβ production (Kabir et al., 2019). Among other proposed neuromodulatory mechanisms of memantine are the reduction of microglial activation, and its anti-inflammatory/anti-apoptotic/antioxidative capabilities (Folch et al., 2018). As crosstalking trajectories are proposed between TGF-β1 and NMDA in astrocytes (Baldwin and Eroglu, 2017), memantine can indirectly mitigate the expression of TGF-β1 and its downstream signaling cascade; p-Smad2 and p21. Memantine was selected herein although acetylcholinesterase inhibitors (AChEIs) are commonly used in the management of AD and are effective for enhancing cholinergic transmission. However, our objective was to address a broader spectrum for the management of cognitive impairment that may not solely depend on cholinergic pathways. Memantine is used for moderate to severe Alzheimer (Dou et al., 2018), which is ideal to target severe cognitive deficits induced by STZ-ICV (Knezovic et al., 2015), compared to donepezil and other AChEIs, which are indicated for milder cases. Memantine also shows the best profile of acceptability among AD patients. Moreover, Memantine has been proven to affect TGF-β, the molecular target of interest in the present work, in many circumstances (Lu et al., 2021; Murakawa-Hirachi et al., 2021). Memantine’s unique mechanism and ability to modulate glutamatergic activity allows for a more comprehensive venue for the neuroprotective effects we aimed to evaluate.

Besides, the observed neurological impact of rosuvastatin could be ascribed to its pleiotropic effects beyond lowering lipid concentration. These lipid-independent pharmacological mechanisms include antioxidant/anti-inflammatory/anti-apoptotic potentials and reduction of Aβ concentration as well as the observed decline in glial burden (Husain et al., 2018a; Husain et al., 2019; Kosowski et al., 2021). All these aptitudes of rosuvastatin might justify its corrective effect on TGF-β1/Smad/p21 trail as well as the histological improvements. Therefore, treatment with rosuvastatin could be a potential new therapeutic strategy for sporadic dementia of AD. In the current study, the failure of rosuvastatin to restore the cognitive derangements in the behavioral tests might seem conflicting with previous studies (Georgieva-Kotetarova and Kostadinova, 2013; Fatima et al., 2017), whereas these inconsistent results might be explained by the dissimilarities in study design or data analysis.

It has been also shown that statins decreased plasma cholesterol, which diminished the cholesterol content of membrane/lipid rafts, disabling the NMDAR and improving the exploratory functions and retention memory of the AD animals as reported earlier (Cruz et al., 2017). However, their effects on CNS cholesterol are less clear as the CNS does not rely entirely on cholesterol from systemic circulation due to limited metabolic turnover and the brain’s inherent capacity to synthesize its own cholesterol. Additionally, the half-life of brain cholesterol is six months up to years, while plasma cholesterol has a half-life of only a few days (Dietschy and Turley, 2004). Thus, rosuvastatin might not be able to disrupt CNS cholesterol balance in a short treatment period despite reducing plasma cholesterol concentrations and hence, longer treatment intervals might be recommended (Cibičková, 2011). Nevertheless, this work presents a novel finding that sub-chronic treatment with rosuvastatin could ameliorate neuronal cell death and protect against future cognitive decline. Such results warrant further investigations to explore the possible effects of statins in AD.

TGF-β1 is an injury-related cytokine that is specifically involved in the regulation of neuronal survival, migration/development of astrocyte and cerebral APP expression (Krieglstein et al., 2002; Siegenthaler and Miller, 2004), which are all abnormal cellular events and pathological hallmarks in AD. TGF-β1 might orchestrate detrimental brain’s response to injury and neurotoxicity via augmenting hippocampal N-methyl-D-aspartate receptor (NMDAR) expression (Bae et al., 2011), and promoting inflammatory/oxidative astrocyte reactivity (Luo, 2022). Previous studies have accentuated the key role of TGF-β1 signaling in senescence regulation by causing oxidative stress-induced activation of the p21 pathway (Samarakoon et al., 2019; Tominaga and Suzuki, 2019). The p21 gene was formerly proven to be upregulated by TGF-β1/Smad pathway (Pardali et al., 2000; Moustakas et al., 2002). The TGF-β1/Smad/p21 pathway is involved in astrocyte senescence in AD and mediates the neurotoxicity properties of astrocytes in AD (Amram et al., 2019). Therefore, in the current investigation, a high correlation was detected between TGF-β1 signaling and the examined markers of cognitive functions, oxidative stress, count of degenerated neurons and gliosis.

In addition, TGF-β may contribute to BBB transporters expression to regulate Aβ clearance. In the current model of AD, ICV-STZ injection resulted in a significant upregulation of RAGE and a down-regulation of ABCA1 and LRP1, which regulate Aβ influx and clearance, respectively, and would favor Aβ accumulation within brain tissue in AD. In addition to stimulating Aβ influx into brain parenchyma, RAGE was found to be a key contributor to AD pathology and Aβ accumulation via other mechanisms; it generates Aβ by enhancing β- and/or γ-secretases activity and triggers Aβ and tau hyper-phosphorylation. Moreover, it contributes to cognition impairment by activating inflammatory response and oxidative stress (Cai et al., 2016). In contrast, ABCA1 is expressed in brain endothelial cells, pericytes, astrocytes, and microglial cells where it enhances ApoE lipidation. Highly lipidated ApoE binds more efficiently, modulates Aβ conformation, diminishes Aβ capacity to aggregate, and facilitates Aβ trafficking into the perivascular space, making Aβ more accessible to LRP1 (Elali and Rivest, 2013). In addition, ABCA1 can positively impact BBB integrity and anti-inflammatory signaling (Lewandowski et al., 2022). Consequently, ABCA1 down-regulation during AD would disrupt BBB integrity, generate pro-inflammatory signaling and decrease ApoE lipidation with subsequent Aβ aggregation and less trafficking into the perivascular spaces to be eliminated by LRP1. Of note, ABCA1 and LRP1 complement the action of each other for Aβ clearance. Besides, LRP1 β-chain was found to positively regulate the expression of ABCA1 in some conditions (El Asmar et al., 2016).

To our knowledge, linking the expression pattern of BBB transporters to TGF-β/Smad signaling in AD was not previously addressed. Data of the present work revealed that TGF-β/Smad signaling is more correlated to LRP1 and ABCA1 expressions, indicating more influence of TGF-β on Aβ efflux rather than on Aβ influx. A study performed by Wyss-Coray et al. (2001) revealed preferential TGF-β1 accumulation and TGF-β1-induced amyloidogenic effects on cerebral perivascular and vascular cells; an effect that highlights potential impact of TGF-β1 to BBB transporters expression.

Correlation between ABCA1 expression and TGF-β sounds to be more related to TGF-β signaling in astrocytes. The astrocyte-targeted overexpression of TGF-β promotes amyloid angiopathy in the frontal cortex and meninges. Astrocyte TGF-β signaling also mediates ApoE neurotoxicity that contributes to Aβ production (Luo, 2022). This may, in part, indicates decreased ABCA1 expression involved in ApoE lipidation and Aβ clearance. Furthermore, the pro-inflammatory effect of TGF-β in AD is mediated by the upregulation of reactivity marker gene complement C3 in an AD-associated astrocyte subpopulation (Grubman et al., 2019). Upregulation of C3 is usually associated with ABCA1 down-regulation in various disease conditions (Alwaili et al., 2012; Suzuki et al., 2012), further indicating possible involvement of TGF-β in regulating ABCA1 gene expression.

The strong negative correlation between TGF-β1 and LRP1 can be explained by the fact that TGF-β1 is a ligand for LRP1 that can mediate its actions through LRP. LRP1 was described to be identical to TGF-βR (V), which is co-expressed with TGF-βR I, II and III. In accordance, LRP1 is required for mediating the growth inhibitory response of TGF-β/Smad signaling through TGF-βR I and II in smooth muscle cells (Boucher et al., 2007). Another proof for the strong TGF-β1/LRP1 correlation is that they are both connected to miRNA-205 regulation; miRNA-205 down-regulates LRP1 expression (Song and Bu, 2009), which is consistent with our results of AD model. As well, miRNA-205 is down-regulated in response to TGF-β (Gregory et al., 2008).

Less correlation was found between TGF-β1 and RAGE expression and most previous studies, in conditions other than AD, correlated RAGE to Smad signaling rather than TGF-β1 cytokine or receptor. For instance, previous *in vitro* studies proved that RAGE–induced ROS generation and pathological effects; including diabetic injury, diabetic nephropathy, accelerated cardiac aging, were mediated by the activation of TGF-β/Smad signaling (Li et al., 2004; Fang et al., 2016). Besides, both RAGE and TGF-β1 share the same regulatory miRNA; as miRNA-185 represses RAGE and TGF-β1 expressions (Jing et al., 2015). This is in line with our results where ICV-STZ injection was coupled with down-regulation of miRNA-185 and RAGE/TGF-β upregulation.

Both memantine and rosuvastatin therapies significantly down-regulated RAGE and upregulated LRP1 and ABCA1 expression reflecting their potential to clear Aβ and matches our results showing a significant decrease in Aβ_1−42_ expression in the hippocampus. Both therapies also significantly affected the miRNAs-185, −205, and − 26 regulating RAGE, LRP1 and ABCA1 expression, respectively. The two drugs more prominently and significantly affected the expression of transporters involved in Aβ efflux; LRP1 and ABCA1, rather than RAGE responsible for Aβ influx as evident from the number of folds increase in transporters expression following treatment compared to its level in untreated rats with induced AD. LRP1 and ABCA1 were also the transporters found to be highly correlated to TGF-β expression as explained earlier in this discussion. Since both drugs were significantly capable of disrupting TGF-β/smad signaling, this could in part explain their greater potential to alter LRP1 and ABCA1 expressions more than RAGE.

The influence of both drugs on ABCA1 was significantly less than their influence on LRP1. A conceivable justification for that order (the effect on LRP1 is greater than on ABCA1) is that LRP1 β-chain takes part in regulating ABCA1 expression (El Asmar et al., 2016), so, LRP1A is supposed to be expressed first to control regulation of ABCA1. In accordance, rosuvastatin showed anticoagulant effect in patients with venous thrombosis by increasing LRP1 expression both *in vitro* and *in vivo* (Paciullo and Gresele, 2019) and high dose rosuvastatin was required to increase ABCA1 transporter in human atherosclerotic plaques (Santovito et al., 2020). Yet, no supporting literature was found showing possible effect of memantine on LRP1 or ABCA1. A different order was observed for the mi-RNAs controlling these transporters and the two drugs more prominently affected miRNA-26, controlling ABCA1, than miRNA-205, controlling LRP1. Possibly, this is because miRNA-26 is not indicative only to ABCA1 level and its expression is affected by other factors and mediators. For example, it was found that miRNA-26 is down-regulated by TGF-*β*1-mediated phosphorylation of Smad2/3, since memantine and rosuvastatin significantly decreased p-smad2, this may contribute to the over-expressed miRNA-26 (Liang et al., 2014). The least effect of both drugs was on RAGE transporter and its regulating miRNA-185. RAGE was also the least transporter correlated to TGF-β/smad signaling. This means that both memantine and rosuvastatin mediates Aβ clearance mainly by modulating BBB transporters at the abluminal surface of brain vessels that contributes to Aβ efflux.

Hence, memantine-rosuvastatin combination showed the maximum reduction in the expression of Aβ, p-Smad2 protein’s, p21gene, MDA content and the maximum increase in the GSH content, which was associated with the lowest number of degenerated neurons and glial cells. Memantine-rosuvastatin regimen can concurrently modulate multiple intertwined systems such as (Aβ/p-Smad2/p21 pathway, NMDA, glutamatergic and redox system (Steullet et al., 2016), in addition to the specific site of action of each drug. Moreover, memantine and rosuvastatin combined therapy showed an additive effect on the expression of ABCA1, LRP1 and RAGE expression. This indicates massive Aβ clearance into blood stream and was reflected on the substantial reduction of Aβ_1−42_ expression in the hippocampus obtained from rats treated with the combination therapy that nearly reached normal values. The combination therapy more significantly increased Aβ efflux out of the brain than decreasing Aβ influx into brain parenchyma by affecting ABCA1/miRNA-26 and LRP1/miRNA-205 expressions more than RAGE/miRNA-185 expression. This effect was similar to either drug alone and was found to be correlated to TGF-β/Smad signaling pathway, which may designate that the effect of these drugs or their combination on Aβ clearance may be in some measure due to their influence on TGF-β/Smad signaling. Thus, the beneficial improvement of AD manifestations in rats by memantine-rosuvastatin combination could be attributed to the effect of both drugs on dampening of the TGF-β1 signaling pathway, which operates a downstream cascade to modulate neuronal integrity. Consequently, a reversal of the overactivity in one or multiple of these aliments by the combined therapy might have beneficial additive potential to restore the function of the whole system (Keshavan et al., 2017).

Moreover, the antioxidant activity of memantine (Kamat et al., 2010; Kumar et al., 2018; Shata et al., 2020), and rosuvastatin (Husain et al., 2018b) has been reported in experimental and preclinical studies (Tanović and Alfaro, 2006; De Felice et al., 2007; Dias et al., 2007; Liu et al., 2013; Sozio et al., 2013). In addition, memantine was reported to attenuate neuronal apoptosis in cerebellar granule cells through an NMDAR-independent mechanism (Weller et al., 1993; Waxman and Lynch, 2005). Likewise, rosuvastatin showed an antiapoptotic potency in ischemic rats (Die et al., 2010). Therefore, rosuvastatin-memantine combination may separately and concurrently target several systems that converge to neuroprotection. Both rosuvastatin and memantine have common mechanisms in attenuating neurodegeneration observed in the brain of AD patients. Thus, the combination of both drugs showed additive/potentiating effects in many aspects making this combination a promising therapeutic regimen in the prevention and treatment of AD.

## Conclusion

The current study settled strong evidence of the major role of TGF-β/Smad signaling pathway in the pathogenesis of AD with high correlation to the impairment in cognitive functions, marked Aβ accumulation, oxidative stress and histopathological abnormalities. Moreover, TGF-β/Smad signaling was found to regulate BBB transporters in charge of Aβ efflux and increased expression of RAGE involved in Aβ influx as well as the expression of their regulatory miRNAs. All these impairments were amended by treatment with memantine, rosuvastatin or their combination. The combination therapy showed an additive/potentiating effect and was superior to either drug alone. In addition, these therapies enhanced Aβ clearance by regulating BBB transporters. Their influence on transporters and miRNAs promoting Aβ efflux was more than their impact on those endorsing Aβ influx, an effect attributable to their regulation of TGF-β/Smad signaling. Taken together, the promising results of the current study open an avenue for newer treatment modalities with good safety profile for AD.

## Supplementary Information

Below is the link to the electronic supplementary material.

ESM 1 (DOCX 14.0 KB)

## Data Availability

All data generated or analyzed during this study are included in this published article and its supplementary information files. The datasets (RNA sequences) analyzed during the current study are available in the GenBank repository, https://www.ncbi.nlm.nih.gov/genbank/, accession numbers are included in Table 1.
