# Supplementary material for: Memantine/Rosuvastatin Therapy Abrogates Cognitive and Hippocampal Injury in an Experimental Model of Alzheimer's Disease in Rats: Role of TGF-β1/Smad Signaling Pathway and Amyloid-β Clearance
Source: J Neuroimmune Pharmacol. 2024 Dec 21;20(1):4. doi: 10.1007/s11481-024-10159-1 (PMC11663181; doi:10.1007/s11481-024-10159-1)
Supplement: Supplementary file 1 — (DOCX 14.0 KB) [file 11481_2024_10159_MOESM1_ESM.docx]

**Supplementary Table 1: Details regarding the animal deaths after the stereotaxic surgery and ICV-STZ injection**

| Rats | Time of death post ICV-STZ injection |
| --- | --- |
| 1^st^ rat | 2 days |
| 2^nd^ rat | 3 days |
| 3^rd^ rat | 3 days |
| 4^th^ rat | 6 days |
| 5^th^ rat | 10 days |
| 6^th^ rat | 13 days |

ICV-STZ: intracerebroventricular injection of streptozotocin
